# Inflammatory Myofibroblastic Tumor of the Hilar Bile Duct: A Case Report and Literature Review

**DOI:** 10.3389/fsurg.2022.928669

**Published:** 2022-09-23

**Authors:** Sheng-qiang Gao, Yong-jin Bao, Jian-sheng Luo

**Affiliations:** Department of Hepatobiliary and Pancreatic Surgery, Affiliated Jinhua Hospital, Zhejiang University School of Medicine, Jinhua, China

**Keywords:** inflammatory, myofibroblastic, tumor, hilar bile duct, case report

## Abstract

**Background:**

Inflammatory myofibroblastic tumor (IMT) is a very rare tumor and occurs seldom in the biliary tract. IMT can occur in any part of the body and in people of any age; however, it most commonly occurs in children or adolescents. Its etiology and pathogenesis are currently unknown. The clinical manifestations of a hilar inflammatory myofibroblastic tumor are atypical, and the imaging examination is nonspecific. The diagnosis is mainly based on histopathology and immunohistochemistry findings, and surgical resection is the preferred treatment method.

**Case Description:**

Herein, we report a rare case of hilar bile duct IMT and review the related literature. Our patient was a 54-year-old woman presenting with a 1-day history of upper abdominal pain as the main clinical symptom. She was misdiagnosed as having cholangiocarcinoma before the surgery. She underwent surgery and was ultimately diagnosed with IMT based on histopathology and immunohistochemistry findings. On 1-year follow-up, no tumor recurrence or related complications were noted.

**Conclusions:**

We hope this case report helps clinicians gain a deeper understanding of biliary IMT of the hilum.

## Introduction

Inflammatory myofibroblastic tumor (IMT) is an exceedingly rare mesenchymal tumor. It is an assembly of differentiated myofibroblastic spindle cells, which commonly presents with the infiltration of large amounts of plasma cells and lymphocytes (1). The lung was the first identified onset site of IMT by Bahadori and Liebow in 1973 (2); thereafter, the mesentery, mediastinum, liver, peritoneum, skin, breast, nerves, bones, and the central nervous system have been reported as onset sites as well (3, 4). The biliary tract is seldom involved, and there are very few reports on the IMT of the biliary tract (5). At present, the pathogenesis of IMT remains elusive. Besides, it is difficult to diagnose given the atypical laboratory and imaging findings, particularly the IMT of the hilum, which is extremely rare. In this context, there is a high risk of misdiagnosis as a malignant lesion. At present, the diagnosis of IMT is mainly based on the histopathology and immunohistology findings, and surgical resection remains the primary treatment. Herein, we report a case of biliary IMT of the hilum and review the literature.

## Case Report

A 54-year-old woman visited our hospital on January 30, 2021 with a 1-day history of pain in the middle upper abdomen. On the day before the admission, she experienced pain in the middle upper abdomen with no obvious precipitating factors. The pain was paroxysmal but not radiating and accompanied yellowing of the skin and eyes. There was no manifestation of fever, vomiting, or diarrhea. When she visited our emergency department, abdominal enhanced CT was performed, wherein dilation of the upper common bile duct (with soft tissue shadows) and intrahepatic bile duct was observed, which led us to suspect a tumor. The patient was then admitted due to an initial diagnosis of a bile duct tumor. Physical examination revealed mild yellowing of the skin and sclera, and no tenderness or palpable mass was noted in the abdomen. The patient reported a previous history of diabetes, which was being managed with oral metformin. She denied having any past medical history of hypertension, heart disease, hepatitis B, tuberculosis, renal disease, pulmonary disease, and smoking and alcohol abuse. She underwent myomectomy three years ago. Routine blood test findings were normal. Blood biochemical profile revealed the following: total bilirubin = 96.1 µmol/L (normal range: 2–25 µmol/L), direct bilirubin = 54.3 µmol/L (normal range: 0–10 µmol/L), indirect bilirubin = 41.8 µmol/L (normal range: 0–10 µmol/L) and alanine aminotransferase (ALT) 616.4 IU/L (normal range: 9–50 IU/L). Tumor markers, including carcinoembryonic antigen (CEA), cancer antigen 19-9 (CA19-9), and alpha-fetoprotein (AFP), were normal. Immunoglobulin G4 (IgG4) was normal as well.

**Figure 1:**
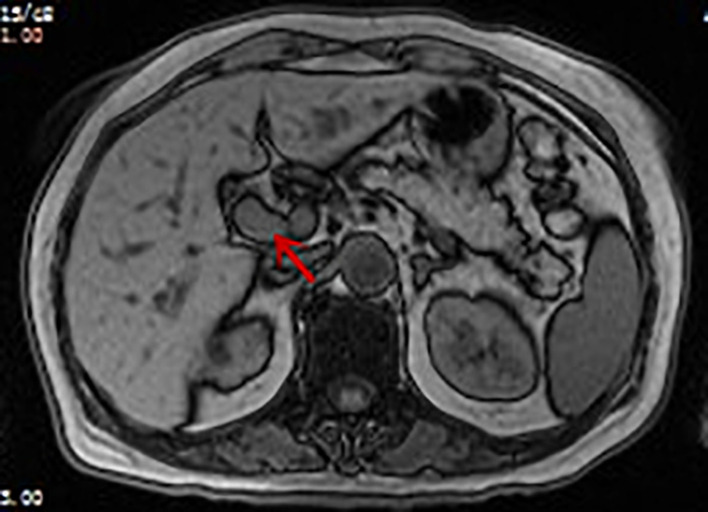
Enhanced MRI image of the patient: soft tissue shadow (indicated by arrow) measuring approximately 1.5 × 1.8 cm can be seen in the hepatic hilum. T1 image: the tumor appears as a slightly low-signal-intensity mass.

**Figure 2:**
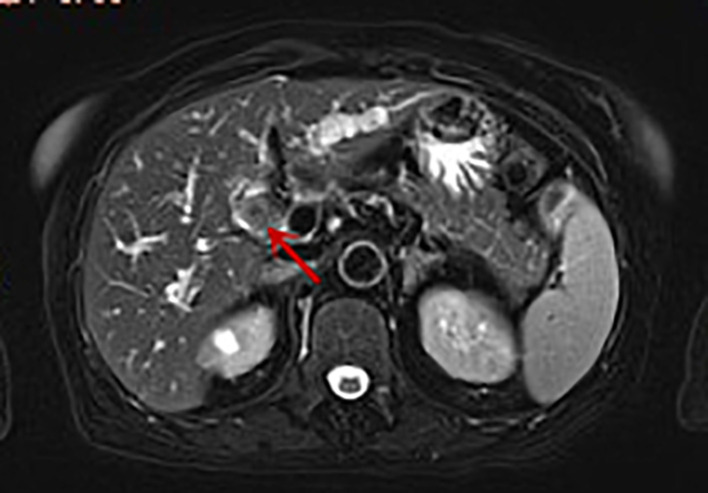
Enhanced MRI image of the patient: soft tissue shadow (indicated by the arrow) measuring approximately 1.5 × 1.8 cm can be seen in the hepatic hilum. T2 image: the tumor appears as a slightly high-signal-intensity mass.

**Figure 3:**
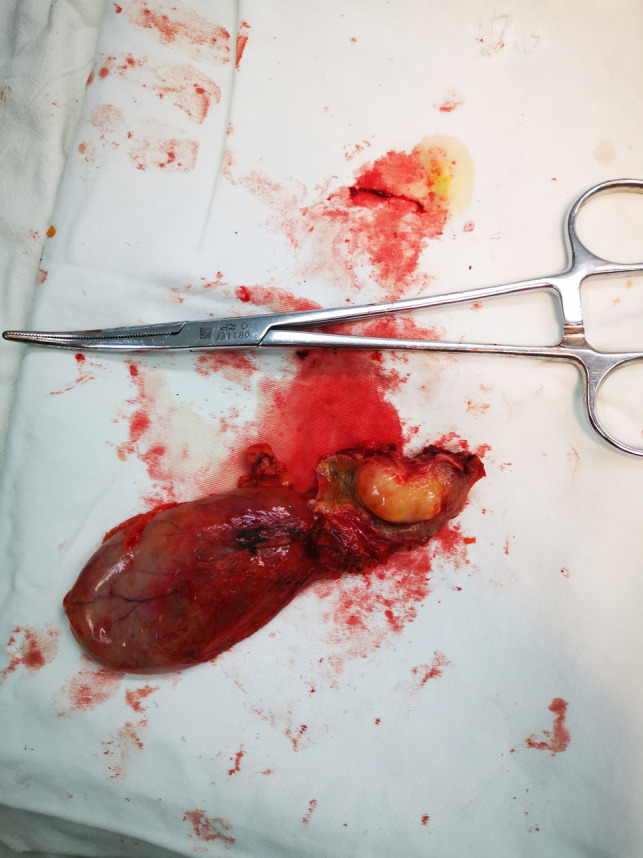
In the patient’s tissue specimen, the tumor is located in the hilar bile duct, the tumor is lipoma-like, and the tumor measures approximately 1.5 × 1.8 cm.

**Figure 4:**
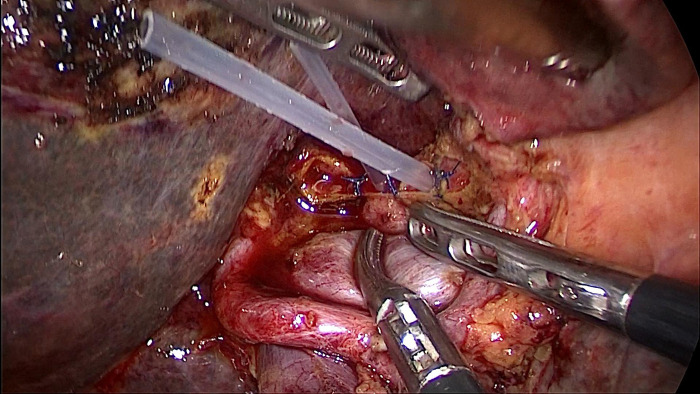
There are four bile duct openings in the hepatic hilum after the resection of the bile duct tumor; the middle two are bile duct openings of the caudate lobe, and the ones on the left and right are the left and right bile duct openings. Two silicone tubes were placed in the two bile ducts of the caudate lobe to prevent closure of the bile ducts.

**Figure 5:**
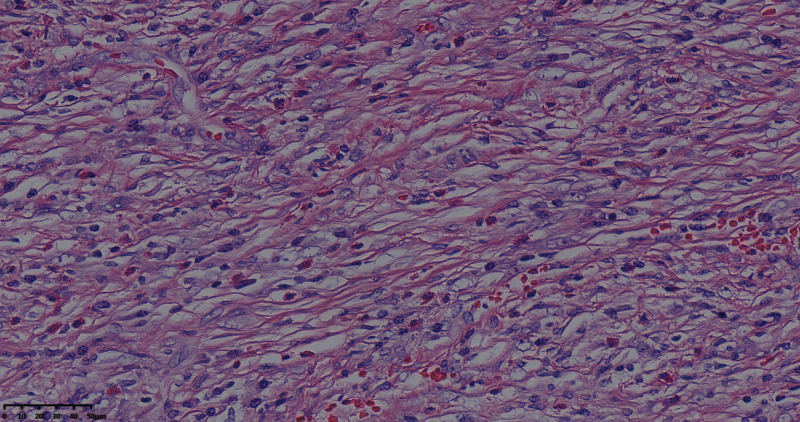
Tumor pathological HE staining pictures: fibroblasts with striae-like proliferation with interstitial collagenization and infiltration of lymphocytes and plasma cells (magnification, ×40).

**Figure 6:**
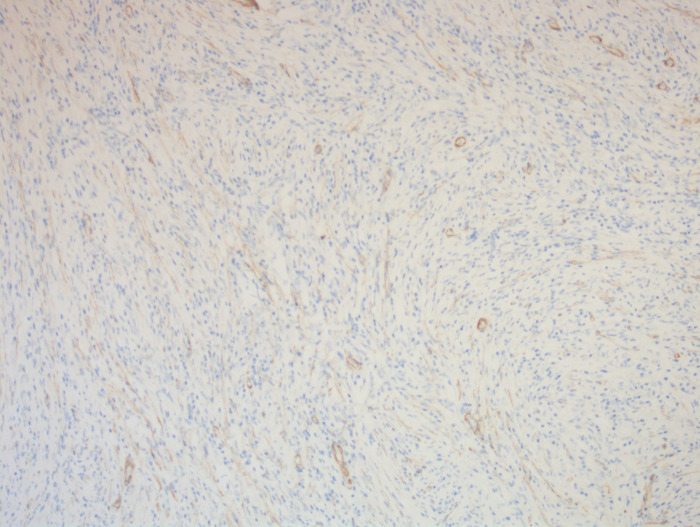
A photomicrograph of immunohistochemistry staining of the lesion. Tumor cells were positive for smooth muscle actin. Smooth muscle of blood vessels acted as an internal positive control. (magnification, ×100).

**Figure 7:**
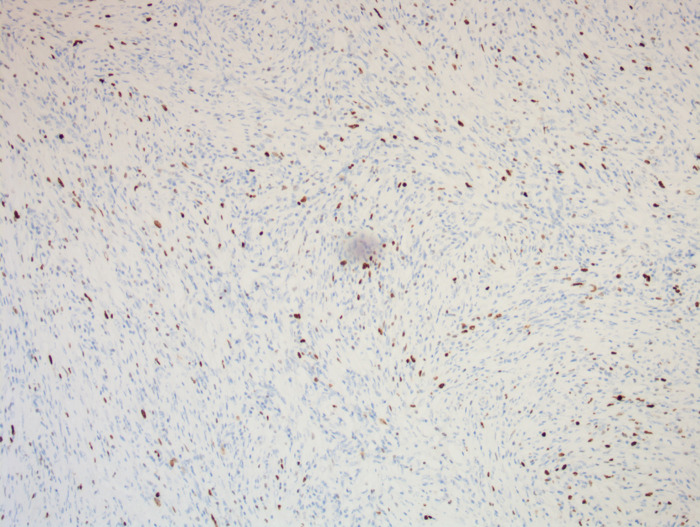
A photomicrograph of immunohistochemistry staining of the lesion. The expression of Ki67 in tumor cells is about 5%. (magnification, ×100).

**Figure 8:**
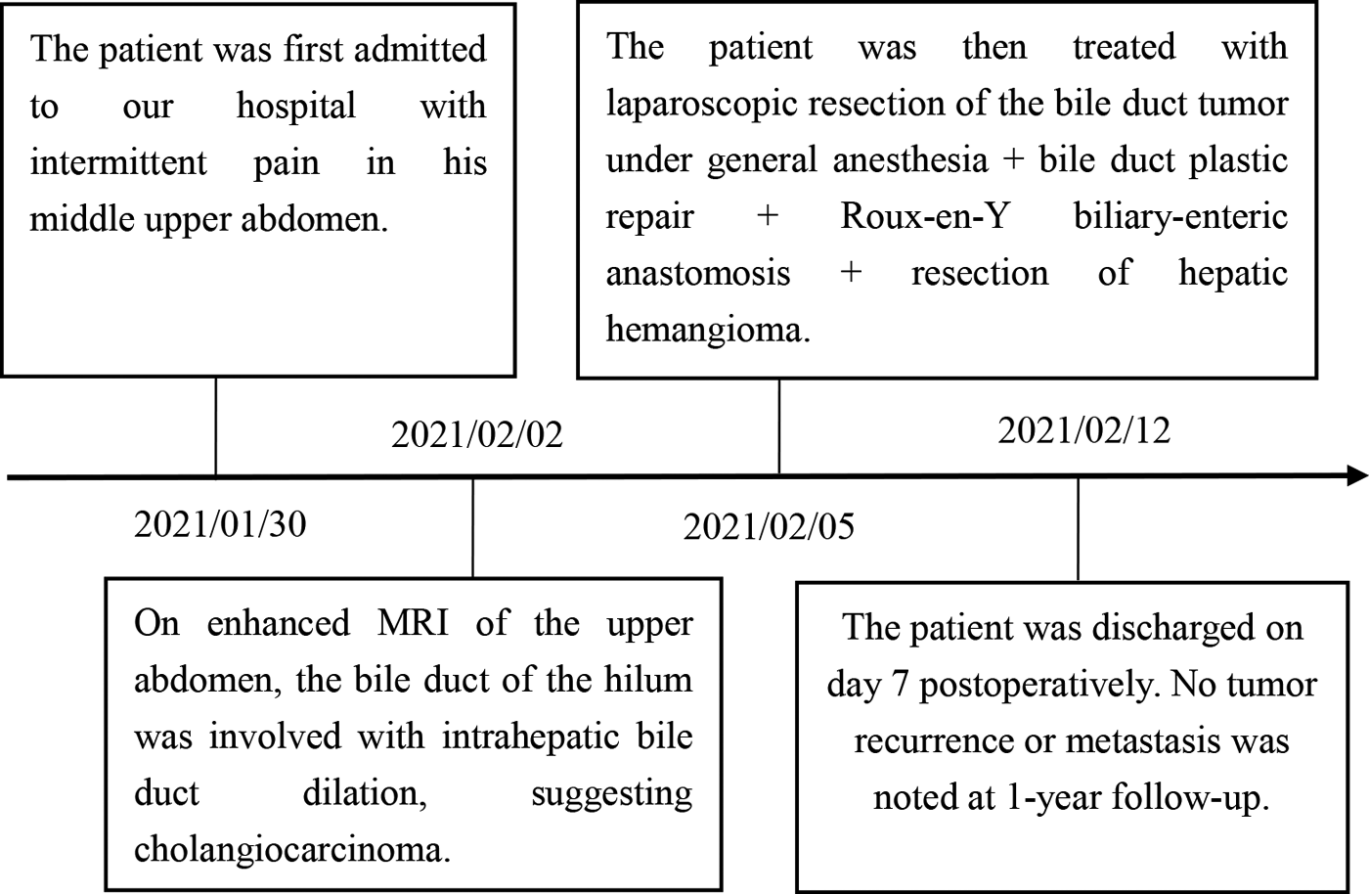
Timeline of symptoms, treatment, and prognosis in our case.

On enhanced MRI of the upper abdomen, the bile duct of the hilum was involved with intrahepatic bile duct dilation, suggesting cholangiocarcinoma. Figure 1 presents the T1 image and Figure 2 presents the T2 image, and the location of the swelling is indicated with a red arrow. In addition, the presence of multiple hepatic masses was noted as well. A small nodule was observed in the right lung. The hepatic mass was considered to possibly have metastatic potential, and its resection was planned. Multiple cysts were observed in both kidneys. Chest CT showed an atelectatic right lung and a ground-glass nodule in the right lower lobe. An initial diagnosis of cholangiocarcinoma of the hilum was made. The patient was then treated with laparoscopic resection of the bile duct tumor under general anesthesia + bile duct plastic repair + Roux-en-Y biliary-enteric anastomosis + resection of hepatic hemangioma. Cholestasis, evident gallbladder swelling, and common bile duct dilation (∼2 cm) were identified during the procedure. In the meantime, two hyperechogenic small nodules (∼0.8 cm) were identified in the liver VIII segment using an ultrasonic probe; these were partially resected using an ultrasonic knife and then pathologically diagnosed as hepatic hemangioma. The bile duct tumor of the hilum was resected along with the gallbladder. The duodenum of the common bile duct was detached, and the lower bile duct was ligated. The hepatoduodenal ligament was dissected. The bile duct above the bifurcation of the right and left hepatic ducts and the two bile ducts of the caudate liver lobes were detached. The separated bile ducts were cut. The tumor was located in the hilum and presented with yellow fat with an intact envelope (Figure 3). Rapid intraoperative pathology indicated negative bile duct margins, suggesting IMT. A Roux-en-Y biliary-enteric anastomosis was performed instead of right hemihepatectomy. First, the four separated bile duct openings were intermittently stitched together with 5-0 prolene sutures (Figure 4). Two silicone tubes were placed into the two bile duct openings of the caudate liver lobe, which supported smooth drainage and prevented the closure of the bile ducts. Bile duct plastic repair was performed laparoscopically as it offers low risk of biliary leakage after sutures; this was followed by the Roux-en-Y biliary-enteric anastomosis. Postoperative pathology identified the hepatic nodule as a hemangioma and revealed that the hilar mass presented with storiform hyperplasia of fibroblasts with interstitial collagen and infiltrating plasma cells and lymphocytes, which are consistent with the presentation of IMT (Figure 5). Besides, margin-negative tests were obtained in all bile ducts. Our surgery achieved R0 resection. Immunohistochemistry revealed smooth muscle actin (weak +), CD34 (–), CD117 (–), Des (–), ki67 (+) <5%, CD21 (–), and CD23 (–). Figure 6 shows positive staining for smooth muscle actin, and Figure 7 shows positive staining for Ki67 in tumor cells. Biliary IMT of the hilum was finally diagnosed. The patient was uneventful after the surgery and had no biliary leakage, abdominal hemorrhage, or infection. The patient was discharged on day 7 postoperatively. No tumor recurrence or metastasis was noted at 1-year follow-up. The timeline of the patient’s symptoms, treatment, and prognosis in our case is shown in Figure 8.

All procedures performed in studies involving human participants were in accordance with the ethical standards of the institutional and/or national research committee(s) and were in compliance with the tenets of the Declaration of Helsinki (as revised in 2013). Written informed consent was obtained from the patient for performing the procedures and publishing the findings as images and text.

## Discussion

IMTs typically comprise myofibroblastic and fibroblastic spindle cells accompanied by an inflammatory infiltrate of plasma cells, lymphocytes, and/or eosinophils. Previously, IMT has been referred to as plasma cell granuloma, inflammatory pseudotumor, inflammatory pseudotumor, inflammatory myofibrohistiocytic proliferation, and inflammatory fibrosarcoma. According to the *WHO Classification of Tumours: Soft Tissue and Bone Tumours, 2020,* IMT was defined as a unique and rare metastatic tumor. It is generally considered benign even though it shares multiple similar characteristics with various invasive malignancies in terms of clinical and radiological manifestations. However, different biological behaviors may be presented by these tumors (6). IMT is defined as an intermediate-grade tumor by WHO and has a propensity for regional recurrence but low incidence of metastasis (7); its incidence does not significantly differ between men and women.

At present, the pathogenesis of IMT is still unclear; however, there are four possible factors that may lead to IMT. (1) Excessive inflammatory reactions caused by trauma or surgical operations and (2) infections caused by *Escherichia coli*, *Staphylococcus* spp., and Gram-positive bacteria (8) have been reported to be associated with IMT development. (3) Some autoimmune diseases, such as primary biliary cirrhosis, thyroiditis, autoimmune pancreatitis, Crohn’s disease, Sjögren’s syndrome, idiopathic thrombocytopenic purpura, and gout, among others can also cause IMT (9). In fact, IgG4 has been reported to be significantly elevated in some patients with IMT, and infiltration of IgG4-positive plasm cells in the focus, majorly including cytokeratin, has been reported (10); however, notably, our case showed no elevation of IgG4. (4) Aberrant ALK (anaplastic lymphoma receptor tyrosine kinase) gene expression has been recently reported to be associated with IMT in some patients. Aberrant NPM-ALK fusion proteins were first reported in anaplastic large cell lymphoma by Morris et al. (11). Aberrant ALK expression has also been reported in IMT, and currently identified ALK fusion genes in IMT include TPM3, TPM4, CLTC, and CARS. It has been established that aberrant ALK gene expression can cause ALK protein abnormality, thus resulting in abnormal proliferation of myofibroblasts and the subsequent advancement of tumorigenesis (12).

The clinical presentation of IMT is generally atypical and mainly associated with the tumor site. In cases that involve the bile duct, the clinical presentation commonly involves painless obstructive jaundice, abdominal pain, weight loss, and fever. The case reported herein presented with jaundice and abdominal pain, which is considered to have resulted from bile duct obstruction because of the tumor. Moreover, the common laboratory findings include increased white blood cell count and C-reactive proteins levels, marginally elevated liver enzymes, normal CEA and serum AFP levels, and marginally increased CA19-9 levels in some patients (5). Our case did not show elevated CA19-9 levels. The increase in CA19-9 levels is not specific to bile duct carcinoma and can occur even in benign cases with liver and gall diseases.

The imaging findings of IMT are generally atypical. On computed tomography (CT), it generally appears as soft tissue masses with varying degrees of enhancement and inhomogeneous density and rarely presents as significantly enhanced necrosis or calcification (13). Comparatively, MRI manifestations are more concentrated on the internal structure of the tumor tissue, which can better indicate the association of the lesion with the important surrounding tissue structures. The lesions are commonly presented as soft tissue masses with unclear boundaries and irregular morphology. The case reported herein had a slightly hypoechogenic mass on T1WI and a slightly hyperechogenic mass on T2WI in MRI scans.

The definitive diagnosis of IMT relies on histopathology and immunohistochemistry findings (14). Some IMT tumors have clear boundaries and pseudocapsules. In cases of recurrence, the boundaries may become irregular, and the tumors can invade the surrounding muscles or bone tissues (15). Recently, ALK has been identified as a favorable diagnostic biomarker for IMT, with approximately 50%–70% of IMT cases showing ALK positivity (15). In addition, high IgG4 expression can also be referenced during the diagnosis of IMT (16, 17). Immunohistochemical studies on T-cell and B-cell subsets may be conducive to differentiating between IMT and lymphoma. Vimentin, smooth muscle actin, and muscle-specific actin are usually locally or diffusely positive in IMT, whereas S100, myosin, CD34, CD117, CD21, CD23, and caldesmon are usually negative. Therefore, immunohistochemical staining is an important approach for the diagnosis and differentiation of IMT.

It is important to distinguish biliary IMT from cholangiocarcinoma. In cases with cholangiocarcinoma, manifestations on a plain CT scan image may include low-density or slightly low-density shadows and iso-density shadows. On enhanced CT scans, arterial phase enhancement of varying degrees is noted, and mild or moderate venous phase enhancement which is inhomogeneous and network-like is present. In addition, manifestations on a plain MRI scan image include T1 signals that are slightly longer relative to the surrounding tissues, T2 signals that are slightly longer or the same length relative to the surrounding tissues, and some T1 signals that are shorter relative to the surrounding tissues in the dilated bile duct. The case reported here was preoperatively misdiagnosed as a cholangiocarcinoma of the hilum and was later confirmed as biliary IMT by postoperative histopathology and immunohistochemistry.

Surgical resection is currently the preferred treatment for biliary IMT as this type of a solid tumor is commonly misdiagnosed as a malignancy under imaging techniques. For patients with contradictions for surgery, other treatments, such as large-dose steroid hormones, nonsteroidal anti-inflammatory drugs, chemotherapy, and radiotherapy, are recommended (18). However, the efficacy of chemotherapeutics remains debatable (19) and needs to be established with further large-scale case studies. A case of IMT was reportedly treated with oral non-steroidal anti-inflammatory drugs or steroids (20). In our patient, the bile duct of the hilum was obstructed by the tumor, and the upper bile duct was dilated, which warranted prompt surgical treatment.

For the past few years, ALK-I has been reported to exhibit high expression in IMT, with a positivity rate of as high as 89% in immunohistochemistry. ALK is also associated with the activation of some other tyrosine kinases because of the regional tumor relapse and metastasis (21). Crizotinib is a tyrosine kinase inhibitor of the ALK receptor; it can suppress the activity of ALK fusion proteins by competing against adenosine triphosphate for binding with the ALK receptor, thereby blocking pathological signaling transmission and subsequently suppressing the spread of tumor cells (22). Crizotinib is an FDA-approved therapeutic drug for ALK-positive non-small cell lung cancer. Moreover, clinical trials have also upheld its effectiveness in treating ALK-positive anaplastic large cell lymphoma and neuroblastoma (23). However, considering the limited therapeutic options, particularly for patients with ALK-negative tumors, Recine et al. reported the role of microenvironmentally targeted agents, such as trabectedin, in the treatment of recalcitrant soft tissue sarcomas (24). The unique antitumor activity of trabectedin comprises not only its cytotoxic activity but also its ability to modulate the tumor microenvironment. There are some recent reports on cases of ALK-negative IMT. Mai et al. reported a case of ALK-negative pulmonary IMT wherein the patient achieved sustained remission after treatment with crizotinib (250 mg, bid) (25). He et al. reported a case of a 68-year-old woman with intrapancreatic IMT and invasion of the spleen and diaphragm (26); the woman was found to be ALK-negative by next-generation sequencing but showed dual amplification of CDK4 and MDM2. Shah et al reported a case of ALK-negative IMT after 12 years of treatment for synovial sarcoma in both lower extremities (27), and the patient had a good prognosis without signs of recurrence at postoperative follow-up. Antonescu et al. found a correlation between the genotype and specific clinical–pathological characteristics of IMT (28). The results of this study confirm that two thirds of IMTs show ALK-related and ROS1-related fusions. Debonis et al. described a case of lung ALK-negative IMT in an 18-year-old female patient who was treated surgically without adjuvant therapy and had no metastasis or recurrence at 2 years of postoperative follow-up (29). Although there is no standard of care for the treatment of IMT, identifying genomic alterations could help redefine the management of patients with ALK-negative disease.

In addition to ALK, other gene rearrangements need to be studied. Besides conventional assays, such as immunohistochemical testing and fluorescence *in situ* hybridization, more extensive molecular assays, such as next-generation sequencing (NGS), may also be required to explore genetic signatures more comprehensively, which could be an important clinical resource (30).

IMT is generally benign and classified as an intermediate-grade tumor characterized by a potential for recurrence and a low incidence of metastasis. Regional recurrence is common, but it is rare to see a local infiltrating or malignant lesion with distant metastasis (31). IMT is more invasive when it initiates in the abdomen or retroperitoneum than other sites and presents with a relatively high risk of multiple recurrences and distant metastasis (31). A study in 2007 revealed that patients with ALK-negative IMT were prone to metastasis (13). In the present case, no evidence of tumor metastasis or recurrence was observed at the 1-year follow-up.

However, this study has some limitations. First, our patient was considered to have bile duct cancer preoperatively, and the liver mass lesion was not clear. At that time, PET-CT should have been considered to more accurately determine the nature of the hilar mass and liver mass and give us a point of reference. Second, the patient did not undergo genetic testing after the operation, and thus, we do not know whether the patient had positive expression of related ALK gene. However, if the patient is identified as having recurrence or metastasis of the disease during the regular postoperative follow-ups, we will recommend the patient to undergo genetic testing.

## Conclusions

To conclude, biliary IMT of the hilum is exceedingly rare and shows atypical clinical presentation and laboratory and imaging findings. Postoperative pathology is the main approach for a definitive diagnosis. Surgery is the preferred and primary treatment of this disease, and most patients recover well postoperatively with a low incidence of recurrence.

## Data Availability

The original contributions presented in the study are included in the article/Supplementary Material, further inquiries can be directed to the corresponding author/s.
